# Protective effect of a novel hydrogel loaded with CM-UCMSCs on vitrified–thawed ovaries during in vitro culture

**DOI:** 10.3389/fbioe.2026.1763994

**Published:** 2026-02-02

**Authors:** Quan Wen, Li Zhao, Ting Wang, Mingjie Bao, Yan Ling, Si Qian, Yixiao Dou, Yabin Lin, Liqun Wang, Gorbachev Dmitrii, Irina Kurzina, Yuan Zhu

**Affiliations:** 1 Obstetrics and Reproductive Health Department, Jiangxi Provincial People’s Hospital, The First Affiliated Hospital of Nanchang Medical College, Nanchang, Jiangxi, China; 2 Nanchang Key Laboratory of Reproductive Genetics and Stem Cells, Nanchang, Jiangxi, China; 3 Medical Department, Shangrao Senior Technical School, Shangrao, Jiangxi, China; 4 Department of Reproductive Health, Jiangxi Provincial Maternal and Child Health Hospital, Nanchang, Jiangxi, China; 5 Department of Clinical Medicine, Nanchang University, Nanchang, Jiangxi, China; 6 Department of Gynecology, Jiangxi Maternal and Child Health Hospital, Nanchang, Jiangxi, China; 7 General hygiene department, Samara state medical university, Samara, Russia; 8 Laboratory of Chemical Technology, National Research Tomsk State University, Tomsk, Russia

**Keywords:** antioxidant, hydrogel, OTCT, umbilical cord mesenchymal stem cells, vitrified-thawed ovaries

## Abstract

Ovarian tissue cryopreservation and transplantation (OTCT) is an important fertility preservation method for female cancer patients; however, its efficacy is limited by post-transplantation ischemia–reperfusion injury, leading to oxidative stress, apoptosis, and fibrosis that impair ovarian reserve and graft function. Mesenchymal stem cell–conditioned medium shows therapeutic potential through paracrine actions, but clinical use is restricted by relatively limited antioxidant capacity and delivery challenges. To address this, an antioxidant-enriched hydrogel (PG-gel) was developed from N-acetylcysteine–modified gelatin and poly (ethylene glycol) succinimidyl succinate, loaded with conditioned medium from umbilical cord mesenchymal stem cells (CM-UCMSCs). This study evaluated the efficacy of PG-gel in protecting vitrified–thawed ovarian tissue during *in vitro* culture. The CM-UCMSCs-loaded PG-gel significantly suppressed intracellular reactive oxygen species generation. The PG + CM-UCMSCs group showed markedly reduced follicle loss, improved follicle morphology, decreased collagen deposition, lower apoptosis (fewer TUNEL-positive cells and reduced caspase-3 expression), diminished oxidative damage (lower 8-OHdG), and enhanced glucose consumption compared with the other culture groups. Transcriptomic analysis revealed downregulation of apoptosis-related genes (e.g., Ddit3, Trib3 and Hmox1) and upregulation of mitochondrial metabolism genes (e.g., Mt-atp8, Mt-nd1 and Mt-cyb). In conclusion, the PG + CM-UCMSCs system provided comprehensive protection to cryopreserved ovarian tissue by mitigating oxidative stress, fibrosis, and apoptosis, likely through regulation of apoptotic signaling and enhancement of mitochondrial energy metabolism, thereby offering a promising strategy to improve OTCT outcomes.

## 1 Introduction

Ovarian tissue cryopreservation and transplantation (OTCT) involve surgically retrieving ovarian tissue before gonadotoxic treatments such as radiotherapy, chemotherapy, or surgery (Chung et al., 2021; Donnez and Dolmans, 2018). The tissue is then cryopreserved. After recovery and ovarian failure, it is thawed and transplanted back into the body. This approach preserves fertility and maintains ovarian endocrine function (Zhang et al., 2017). To date, more than 200 babies have been born worldwide using this technique, with births increasing sharply in recent years, and thousands of girls and young women have undergone ovarian tissue cryopreservation (Dolmans et al., 2021; Yding Andersen et al., 2019). The use of OTCT for fertility preservation is expected to benefit a growing number of female cancer patients and individuals at high risk of benign ovarian failure.

In the early stage of cryopreserved ovarian tissue transplantation, the graft undergoes an ischemic and hypoxic period of approximately 3–5 days due to the absence of vascular anastomosis (Van Eyck et al., 2009; Xu et al., 2022). This process induces oxidative stress, apoptosis, and fibrosis, which collectively impair ovarian function (Dolmans et al., 2021). Studies have shown that 50%–90% of ovarian reserve function is lost before complete revascularization and that the fibrotic surface area can reach 40%–70% after 24 days (Dolmans et al., 2021; Nisolle et al., 2000). Such impairment markedly reduces the efficacy of clinical applications. Consequently, effective strategies are needed to preserve the functionality of transplanted ovaries.

Mesenchymal stem cells (MSCs) are widely used in tissue regeneration and injury repair, primarily through the secretion of diverse growth factors (Wang et al., 2020). By collecting the supernatant from MSC cultures and subjecting it to filtration and ultraconcentration, a conditioned medium (CM-MSCs) rich in MSC secretions can be prepared (Khalil et al., 2019). Studies have shown that CM-MSCs promote cell proliferation, reduce apoptosis, and exert antioxidant and anti-inflammatory effects (Asadi-Golshan et al., 2021; Sahraei et al., 2022; Un et al., 2023). In clinical research, umbilical cord mesenchymal stem cells (UCMSCs) are the most widely used stem cells, and their exosomes demonstrate antioxidant, antiapoptotic, anti-inflammatory, and tissue-regenerative effects in various diseases (Stavely and Nurgali, 2020; Yang et al., 2023). The conditioned medium of UCMSCs (CM-UCMSCs) is rich in components with anti-ROS activity, such as exosomes, soluble proteins, cytokines, and growth factors. However, CM-UCMSCs exhibit a relatively limited intrinsic antioxidant capacity. Furthermore, conventional delivery methods, such as direct tissue injection or intracavitary infusion, face significant limitations, including transient therapeutic effects, poor retention of active components, and the necessity for repeated administrations.

Hydrogel is a three-dimensional network material formed by the cross-linking of hydrophilic polymer compounds through physical or chemical means, exhibiting excellent permeability to water-soluble substances and a high drug-loading capacity (Xue et al., 2022). Hydrogel enables excellent *in-situ* retention and realizes the gradual release of exosomes, cytokines, and other biomolecules. Numerous studies have shown that hydrogels loaded with cytokines hold broad application prospects in the biomedical field (Huang et al., 2015; Kim and Tabata, 2016; Zhang et al., 2018). Our team has developed a succinimide ester-based hydrogel (SEgel) using poly (ethylene glycol) succinimidyl succinate (PEG-SS) and gelatin. This hydrogel demonstrates a degradation period of approximately 7–10 days and possesses antioxidant properties (Feng et al., 2023; Wang et al., 2022). Furthermore, its antioxidant capability can be substantially enhanced by sulfhydryl modification (Feng et al., 2025). Therefore, this hydrogel is expected to serve as an excellent carrier for loading CM-UCMSCs and for localized application in transplanted ovarian tissues, thereby providing sustained protective effects and improving the clinical outcomes of OTCT.

**SCHEME 1:**
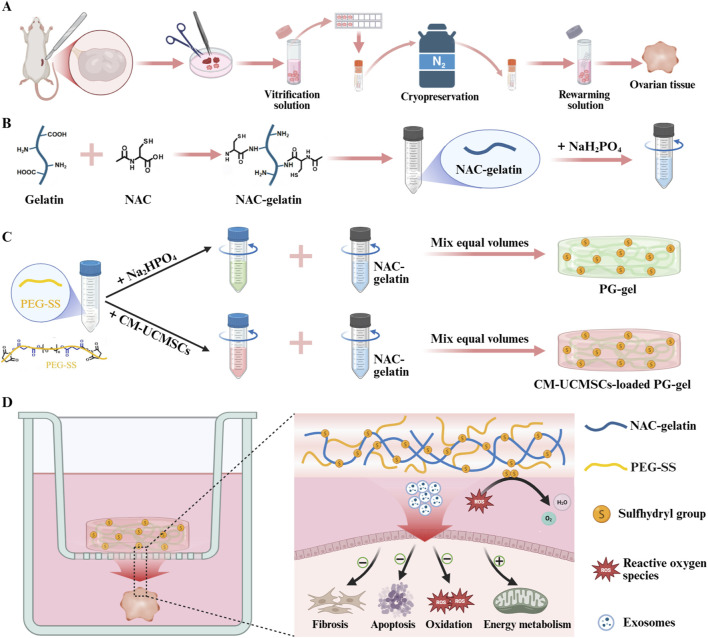
Schematic illustration of the novel hydrogel loaded with CM-UCMSCs for protecting vitrified-thawed ovaries during *in vitro* culture. **(A)** Ovary collection, cryopreservation, and thawing. **(B)** Preparation of the NAC-gelatin solution. **(C)** The PG-gel introduces a high density of sulfhydryl groups and can serve as a three-dimensional scaffold for loading CM-UCMSCs. **(D)** During the *in vitro* culture of the vitrified-thawed ovary, the CM-UCMSCs-loaded PG-gel utilizes its sulfhydryl groups and the sustained release of bioactive factors (e.g., exosomes) to provide comprehensive protection. This protective effect mitigates oxidative stress, fibrosis, and apoptosis, while enhancing energy metabolism.

In this study, we utilized vitrified and subsequently thawed ovarian tissues as the main experimental material (Scheme 1A).We modified gelatin with N-acetylcysteine (NAC) to introduce sulfhydryl groups (–SH) and enhance antioxidant capacity (Scheme 1B). The NAC-modified gelatin (NAC-gelatin) was then crosslinked with PEG-SS to form a novel antioxidant hydrogel, termed PG-gel (Scheme 1C). PG-gel loaded with CM-UCMSCs (Scheme 1C) was subsequently applied to an *in vitro* culture system of cryopreserved–thawed ovarian tissue to evaluate the reparative and protective effects of this hydrogel system on ovarian tissue. The results demonstrate that CM-UCMSCs-loaded PG-gel, through its sulfhydryl groups and gradual release of bioactive factors, offers broad protection by reducing oxidative stress, fibrosis, and apoptosis, and boosting energy metabolism (Scheme 1D). Based on the established hydrogel platform, the main novelty of the current work lies in the first application of PG-gel loaded with CM-UCMSCs to an *in vitro* culture system of vitrified-thawed ovarian tissue. This has enabled the construction of a synergistic “hydrogel-cellular component” composite system, and the systematic evaluation of its reparative and protective effects on ovarian tissue.

## 2 Materials and methods

### 2.1 Preparation of PG-gel and CM-UCMSCs/DMEM-loaded PG-gel

The synthesis method of PEG-SS was identical to that reported in our previous article (Wang et al., 2022). Briefly, polyethylene glycol (PEG; molecular weight: 6000 Da; Meryer, Shanghai, China), 4-dimethylaminopyridine (DMAP; Aladdin, China), and succinic anhydride (SA; Shaoyuan, China) were dissolved in methylene chloride and reacted at room temperature for 12 h. The mixture was washed four times with saturated sodium chloride solution and 1 M hydrochloric acid to remove impurities. Precipitation with anhydrous ether, followed by filtration and vacuum drying, yielded a white powdery carboxyl-terminated PEG. Subsequently, carboxyl-terminated PEG, 1-(3-dimethylaminopropyl)-3-ethylcarbodiimide hydrochloride (EDC; Meryer, China), and N-hydroxysuccinimide (NHS; Meryer, China) (molar ratio 1:2:5) were dissolved in methylene chloride and reacted under the same conditions for another 12 h. The same separation and purification procedures were then repeated to obtain PEG-SS.

NAC-gelatin was synthesized using a two-step method based on a previous study (Gong et al., 2024). First, porcine skin gelatin (Type A; Sigma-Aldrich, Germany) and anhydrous ethylenediamine (Damao, China) were dissolved in phosphate-buffered saline (PBS) at room temperature. After visible changes occurred, the pH was adjusted to 5.5 with HCl. EDC was then added, and the reaction proceeded for 24 h. The product was dialyzed (8000–14,000 kDa) against deionized water for 3 days and freeze-dried to yield amino gelatin (Agelatin). In the second step, Agelatin, NAC (Solarbio, China), and EDC were dissolved in PBS. The pH was again adjusted to 5.5, and the reaction proceeded for 24 h in the dark. Upon completion of the reaction, the solution was dialyzed against deionized water for 3 days and then freeze-dried to obtain purified NAC-gelatin.

The structures of PEG-SS and NAC-gelatin were confirmed by ^1^H NMR spectroscopy (JEOL/JNM-ECZ400S/L1, 400 MHz, Japan). NAC-gelatin was sterilized and dissolved at 20 wt% in 0.1M NaH_2_PO_4_ solution. The solution was heated in a 60 °C water bath until fully dissolved and then maintained at 37 °C for 30 min. PEG-SS was dissolved at 10 wt% in 0.1M Na_2_HPO_4_ solution. Equal volumes of the two solutions were mixed to form the final PG-gel. Substituting Na_2_HPO_4_ solution with DMEM or CM-UCMSCs produced DMEM-loaded PG-gel and CM-UCMSCs–loaded PG-gel, respectively.

### 2.2 Morphology and elemental analysis

Material characterization was performed using scanning electron microscopy with energy-dispersive spectroscopy (SEM-EDS). Plain PG-gel and CM-UCMSCs–loaded PG-gel were frozen at −80 °C and lyophilized. The adhesive disk samples were sputter-coated with gold and examined using SEM-EDS (ZEISS EVO10, Germany). Relative atomic and weight percentages (wt%) of carbon, oxygen, and sulfur were obtained from the EDS spectra.

### 2.3 ABTS radical scavenging assay

Equal volumes of 7.4 mM ABTS (2,2′-Azinobis-(3-ethylbenzthiazoline-6-sulphonate)) and 2.45 mM potassium persulfate solutions were mixed and incubated in the dark at 25 °C for 12 h to generate the ABTS radical stock solution. The resulting solution was then diluted with deionized water to adjust the absorbance to 0.2 at 734 nm. Subsequently, 20 mg of PG-gel, DMEM-loaded PG-gel, CM-UCMSC-loaded PG-gel, and an equal volume of DMEM medium and CM-UCMSCs were mixed with the diluted ABTS radical solution. The mixtures were incubated in the dark at 37 °C for 1 h. The absorbance was measured at 734 nm using a multi-scan spectrophotometer (SuPerMax 3100, Shanpu, China). The ABTS radical scavenging activity was calculated according to the following equation: ABTS radical scavenging rate (%) = (A_0_ - A1)/A_0_ × 100, where A_0_ represents the absorbance of the control (0 mg), and A_1_ represents the absorbance of the sample.

### 2.4 Antioxidant capacity assay

The antioxidant capacity of novel hydrogel was assessed by measuring reactive oxygen species (ROS) levels using the peroxide-sensitive fluorescent probe 2′,7′-dichlorofluorescein diacetate (DCFH-DA). The human ovarian granulosa cell line (KGN) was obtained from Wuhan Huayan Biotechnology Co., Ltd., Wuhan, China. Cells were cultured in DMEM/F12 medium (Procell, Wuhan, China) supplemented with 10% fetal bovine serum (FBS; Excell Bio, Shanghai, China) and 1% antibiotics (Aladdin, Shanghai, China) and maintained at 37 °C in a 5% CO_2_ atmosphere. KGN cells in the logarithmic growth phase were suspended at 2.5 × 10^5^ cells/mL, and 2 mL was seeded into each well of a six-well plate and incubated overnight under the same conditions. Cells were divided into the following groups: the blank group, which received no additives; the H_2_O_2_ group, treated with 10 μM H_2_O_2_; the CM-UCMSCs group, treated with 10 μM H_2_O_2_ and 0.25 mL of CM-UCMSCs; the PG + DMEM group, treated with 10 μM H_2_O_2_ along with a PG-gel placed in the chamber above the culture dish; and the PG + CM-UCMSCs group, treated with 10 μM H_2_O_2_ plus a CM-UCMSCs–loaded PG-gel positioned in the chamber above the culture dish. After 24 h of intervention, cells were digested, collected, and washed with PBS. Following the DCFH-DA assay kit instructions (Beyotime, Shanghai, China), DCFH-DA was used as a fluorescent probe to detect intracellular ROS by flow cytometry with excitation at 488 nm and emission at 530 nm.

### 2.5 Adhesion strength test

An adhesive strength test was conducted using porcine skin cut into 25 mm by 10 mm rectangles. PG-gel, DMEM-loaded PG-gel, and CM-UCMSCs-loaded PG-gel (40 μL each) were applied to one skin piece. The skin pieces were then overlapped, pressed for 1 min, and incubated at 37 °C for 30 min. Three samples were tested for each formulation, and adhesive strength was measured using an electronic universal testing machine (CMT1103, Zhuhai Sansi Test Equipment Co., Ltd., China).

### 2.6 Ovary collection, cryopreservation, and thawing

The experimental animals were female SD rats, 8 weeks old and weighing 180–200 g, obtained from Weitong Lihua (Zhejiang, China) Laboratory Animal Technology Co., Ltd. All protocols were approved by the Nanchang University Institutional Animal Care and Use Committee (approval code: NCULAE-20250905001). Prior to experimentation, all rats had stable estrous cycles confirmed by vaginal smears. After sacrifice by cervical dislocation, ovaries were excised and placed in L15 medium (Solaibao, Beijing, China), with residual oviductal tissues and fat removed. The tissues were trimmed to 2 × 1 × 1 mm. For vitrification, the Surviving Tissue Cryopreservation and Resuscitation Kit (Huicun Medical Company, Shanghai, China) was used. According to the kit instructions, ovarian tissue was immersed in V1 solution at room temperature for 10 min, transferred to V2 solution for 20 min, and then cryopreserved in liquid nitrogen. After 1 month, tissue was retrieved, immersed in T1 solution at 37 °C for 3 min, and sequentially transferred to T2 and T3 solutions at room temperature for 10 min each, preparing it for subsequent experiments.

### 2.7 Preparation of CM-UCMSCs

Umbilical cords were collected from full-term healthy neonates. Residual blood was washed away with PBS, and cords were cut into 2-cm segments. The cords were longitudinally dissected, and the arteries, veins, and Wharton’s jelly were removed. The remaining tissue was cut into 1.0 mm^3^ pieces and attached to the walls of culture flasks. Ten milliliters of DMEM/F12 medium (VivaCell, Shanghai, China) containing 10% fetal bovine serum and 1% Penicillin/Streptomycin Solution 100X (Solaibao, Beijing, China) was added, and the flasks were placed in a 37 °C incubator with 5% CO_2_. When cells reached 80% confluence, they were passaged. Conditioned medium was collected from UCMSCs cultured for three to four generations. When third to fourth generation UCMSCs reached 80% confluence, the medium was replaced with serum-free DMEM/F12, and cells were cultured for another 48 h. The supernatant was then collected, centrifuged at 240 ×*g* for 10 min at 4 °C to obtain the CM-UCMSCs, which was then aliquoted and stored at −80 °C.

### 2.8 Grouping and *in vitro* culture system

Six groups were established in the experiment. For culture groups, ovarian tissue was placed at the bottom of a dish with culture medium added and then incubated at 37 °C with 5% CO_2_ for 2 days. Under effective intervention conditions, a short-term 2-day culture of vitrified-thawed ovarian tissue can significantly enhance functional indicators such as angiogenic capacity and stromal cell proliferation, indicating that this culture period is suitable for assessing the early protective effects of the system (Kang et al., 2016). The groups were as follows: (1) DMEM group, cultured in DMEM medium; (2) CM-UCMSCs group, cultured in CM-UCMSCs; (3) PG + DMEM group, with DMEM medium and a DMEM-loaded PG-gel in the upper chamber; and (4) PG + CM-UCMSCs group, with CM-UCMSCs medium and a CM-UCMSCs–loaded PG-gel in the upper chamber. In these groups, the PG-gel in the upper chamber slowly diffused into the culture system. The remaining two groups, without *in vitro* culture, were the control group (fresh ovarian tissue) and the vitrified–thawed group (tissue that had undergone vitrification and thawing).

### 2.9 Haematoxylin-eosin (HE) staining for counting follicles and masson trichrome staining for quantifying collagen deposition

Ovarian tissues from each group were fixed in 4% formaldehyde and embedded in paraffin. The blocks were cut into continuous 5 μm sections and stained with hematoxylin and eosin. Sections were observed under a light microscope (Olympus) at 200× magnification for follicle counting. Follicles were classified as primordial (oocytes surrounded by a single flat layer of granulosa cells (GCs)), primary (single layer of cuboidal GCs), secondary (two or more layers of GCs without an antrum), and antral (presence of an antrum) (Marschalek et al., 2021). Follicles showing oocyte nuclear degeneration, wrinkled nuclear membranes, vacuoles, or GC degeneration—including shrinkage, pyknosis, and karyorrhexis—were identified as atretic (Shi et al., 2009). Cystic follicles were defined as expanded follicles filled with follicular fluid and lined by one to four layers of round to flat GCs (Shi et al., 2009). Based on morphology, follicles were categorized as healthy (primordial, primary, secondary, and antral) or unhealthy (atretic and cystic). For Masson trichrome staining, sections were processed with a Masson trichrome kit (Servicebio, Wuhan, China) according to the manufacturer’s instructions.

Masson trichrome staining renders muscle fibers red and collagen fibers blue. For each ovarian tissue sample, five random high-magnification fields (400×) were selected, and the collagen deposition area ratio (collagen fibrosis area/total tissue area) was quantified using ImageJ software (version 1.54).

### 2.10 Immunofluorescence and immunohistochemistry

To assess ovarian apoptosis, tissues from each group were subjected to TUNEL staining with a commercial apoptosis detection kit (Servicebio, Wuhan, China) according to the manufacturer’s instructions. Apoptotic signals appeared as green fluorescence, while nuclei were counterstained with DAPI (blue). For quantitative analysis, five random fields per ovary were examined at 400× magnification under a confocal laser scanning microscope, and the average apoptotic rate was calculated using ImageJ software.

Immunohistochemical staining was performed to detect the apoptosis marker caspase-3 and the oxidative DNA damage marker 8-hydroxy-2′-deoxyguanosine (8-OHdG). Positive staining appeared as brown coloration in the cytoplasm or nucleus. For each ovarian tissue sample, five random high-magnification fields (400×) were selected, and the caspase-3 positive rate and mean optical density (MOD) of 8-OHdG were analyzed using ImageJ software.

### 2.11 Glucose assay

To assess the energy metabolism of ovarian tissue cells, glucose content in the culture medium was measured before and after *in vitro* culture for the four cultured groups, and the difference was calculated. Glucose concentration was determined using a glucose assay kit (Huili Biotech, Changchun, China) and an automatic biochemistry analyzer (Chemray 240, Lei Du Life Scientific and Technical Corporation, Shenzhen, China).

### 2.12 RNA sequencing

Three groups were selected for high-throughput RNA sequencing: the DMEM group, the PG + CM-UCMSCs group, and the control group (fresh ovarian tissue), with three samples per group. Sequencing was performed on the Illumina HiSeq platform using the Illumina TruSeq™ RNA sample prep kit for library construction. All RNA-sequencing data from this study have been deposited in the NCBI-SRA database (https://www.ncbi.nlm.nih.gov/sra/) under project number PRJNA1327384 and are publicly available. Raw paired-end reads were trimmed and quality-checked with fastp using default settings. Clean reads were then aligned to the reference genome in orientation mode with HISAT2, and mapped reads from each sample were assembled in a reference-based manner using StringTie. Differentially expressed genes (DEGs) between groups were identified with EdgeR. Significance thresholds were set at false discovery rate (FDR) < 0.05 and |log2FC| ≥ 1. Functional enrichment analysis, including GO and KEGG, was conducted to identify DEGs significantly enriched in GO terms and metabolic pathways at a Bonferroni-corrected P ≤ 0.05 compared with the whole-transcriptome background. GO functional enrichment and KEGG pathway analyses were performed with Goatools and KOBAS, respectively.

### 2.13 Statistics analysis

All results are expressed as mean ± standard deviation (SD). Statistical analyses were conducted with Statistical Package for the Social Sciences software version 26.0 (IBM), and significance was set at P < 0.05. Two-tailed independent samples t-tests were used for pairwise comparisons, and analysis of variance followed by Bonferroni’s *post hoc* test was applied for multiple comparisons.

## 3 Results

### 3.1 Antioxidant capacity and adhesion properties of PG-gel

**FIGURE 1:**
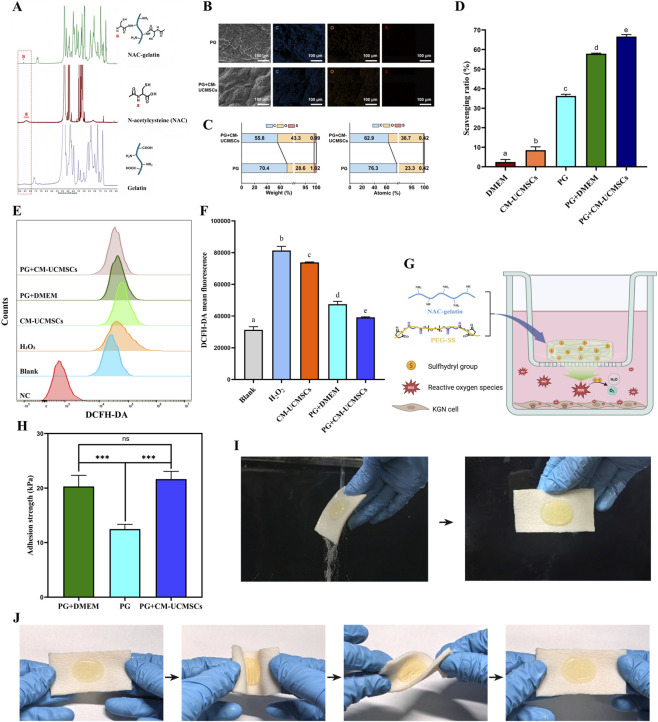
Antioxidant capacity and adhesion properties of PG-gel. **(A)** NAC-gelatin was synthesized by introducing sulfhydryl groups from N-acetylcysteine into the gelatin structure. **(B)** SEM images with corresponding EDS elemental maps and **(C)** EDS elemental composition (C, O, S) of PG-gel and CM-UCMSCs–loaded PG-gel. **(D)** Scavenging rate of ABTS^+^ by DMEM medium, CM-UCMSCs, PG-gel, DMEM-loaded PG-gel, and CM-UCMSCs-loaded PG-gel (mean ± SD, n = 3). **(E)** Flow cytometry of intracellular ROS generation and **(F)** quantitative assessment of ROS levels (mean ± SD, n = 3). NC = negative control. In panels D and F, statistical differences (P < 0.05) were indicated by lowercase letters: bars sharing the same letter were not significantly different, whereas those without the same letter differ significantly. **(G)** Schematic of PG-gel scavenging intracellular ROS in KGN cells. **(H)** Adhesion strength of PG-gel, DMEM-loaded PG-gel, and CM-UCMSCs-loaded PG-gel (mean ± SD, n = 3, ns: not significant, ***p < 0.001). Photographs showed the adhesion property of CM-UCMSCs-loaded PG-gel on porcine skin under water flow **(I)** and torsion **(J)**.

To enhance the antioxidant properties of PG-gel, gelatin was modified with NAC by introducing sulfhydryl groups, producing NAC-gelatin. The ^1^H-NMR spectrum of the modified gelatin showed a new peak at 8.5–8.7 ppm, corresponding to the NH group of the NAC molecule (labeled “a” in Figure 1A), which confirms the successful modification of gelatin with NAC. SEM-EDS analysis showed that PG-gel developed a porous structure after loading with CM-UCMSCs (Figure 1B). Carbon, oxygen, and trace sulfur were detected in both pure PG-gel and CM-UCMSCs–loaded PG-gel. Sulfur content was 1.02 wt% in PG-gel and 0.99 wt% in CM-UCMSCs–loaded PG-gel, with an atomic percentage of 0.42% in both samples (Figure 1C). To evaluate the antioxidant capacity, an *in vitro* ABTS radical scavenging assay was conducted. Results showed that the CM-UCMSCs-loaded PG-gel exhibited the most potent free radical scavenging ability, followed by the DMEM-loaded PG-gel, PG-gel, CM-UCMSCs, and DMEM medium, in descending order. Moreover, statistical significance (P < 0.05) was observed in all pairwise comparisons among the five groups (Figure 1D). Antioxidant capacity was assessed by measuring intracellular ROS levels in KGN cells with the DCFH-DA fluorescent probe. After 24 h of treatment, ROS production was evaluated. The ROS levels, ranked from highest to lowest, were observed in the H_2_O_2_ group, the CM-UCMSCs group, the PG + DMEM group, the PG + CM-UCMSCs group, and the blank group. Statistically significant differences were noted between each pair of groups (Figures 1E,F). These findings suggest that both PG-gel and CM-UCMSCs exhibit a capacity to inhibit H_2_O_2_-induced intracellular ROS generation, and this inhibitory effect is significantly amplified when the two are used in combination. Crosslinking with PEG-SS yields PG-gel from NAC-gelatin, which introduces a high density of sulfhydryl groups. These sulfhydryls serve as a scavenger by neutralizing the ROS generated from KGN cells into harmless products like H_2_O and O_2_, thus effectively mitigating oxidative stress injury (Figure 1G). PG-gel loaded with CM-UCMSCs can also release bioactive factors (e.g., exosomes) that participate in scavenging ROS.

Effective tissue adhesion plays a crucial role in maintaining the stability of adhesive hydrogels, thereby enhancing the mechanical integrity of the materials to which they are applied. The adhesion strength test results indicated that the adhesion strength values for PG-gel, DMEM-loaded PG-gel, and CM-UCMSCs-loaded PG-gel were 12.49 ± 0.86 kPa, 20.31 ± 2.04 kPa, and 21.66 ± 1.41 kPa, respectively. Notably, both the DMEM-loaded PG-gel and the CM-UCMSCs-loaded PG-gel demonstrated significantly greater adhesion strength than the PG-gel (both P < 0.001) (Figure 1H). The CM-UCMSCs-loaded PG-gel kept stable on the porcine skin under water flow and torsion, further demonstrating its excellent adhesiveness (Figures 1I,J).

### 3.2 PG-gel loaded with CM-UCMSCs protects follicles and inhibits ovarian fibrosis

**FIGURE 2:**
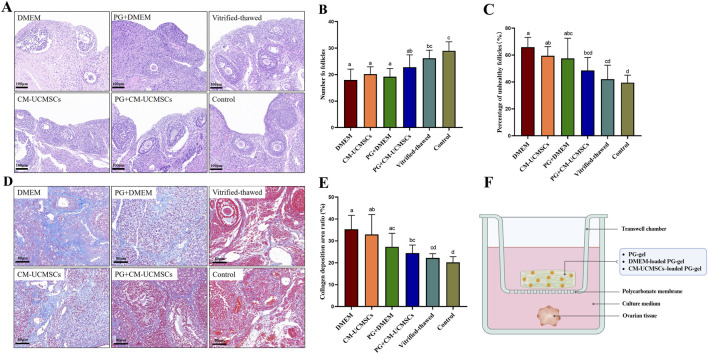
PG-gel loaded with CM-UCMSCs protected follicles and inhibits ovarian fibrosis *in vitro*. Ovarian sections from each group were subjected to HE staining (**(A)**; scale bar = 100 µm), showing follicle counts **(B)** and percentages of unhealthy follicles **(C)** across six groups. Masson’s trichrome staining was also performed (**(D)**; scale bar = 80 µm), with collagen fiber deposition area ratios compared **(E)**. Data are mean ± SD, n = 5 (biological replicates). Statistical differences (P < 0.05) were indicated by lowercase letters: bars sharing the same letter were not significantly different, whereas those without the same letter differ significantly. **(F)** The schematic diagram of the ovarian tissue *in vitro* culture.

Ovarian tissues from each group underwent HE staining (Figure 2A), and follicles were classified and counted. Total follicle numbers were significantly reduced in the DMEM, CM-UCMSCs, and PG + DMEM groups compared with those in the vitrified–thawed and control groups (Figure 2B; P < 0.05). In contrast, the PG + CM-UCMSCs group showed less follicle loss, with a significant reduction only when compared with the control group (Figure 2B; P < 0.05). The percentage of unhealthy follicles was higher in the PG + CM-UCMSCs group than in the vitrified–thawed (P > 0.05) and control groups (P > 0.05) but lower than in the CM-UCMSCs (P > 0.05), PG + DMEM (P > 0.05), and DMEM groups (P < 0.05) (Figure 2C). The rates of unhealthy follicles were also lower in the CM-UCMSCs (P > 0.05) and PG + DMEM groups (P > 0.05) than in the DMEM group (Figure 2C). HE histology indicated that PG-gel or CM-UCMSCs in the *in vitro* ovarian culture system reduced unhealthy follicle formation and that the combined PG-gel loaded with CM-UCMSCs significantly protected follicles.

To compare ovarian fibrosis among groups, Masson staining was performed (Figure 2D), followed by relative quantitative analysis of collagen fiber content. The collagen fiber deposition area ratio was significantly lower in the PG + CM-UCMSCs, vitrified–thawed, and control groups than in the DMEM group (Figure 2E). Ratios were also lower in the CM-UCMSCs and PG + DMEM groups than in the DMEM group, though not statistically significant (Figure 2E). These findings suggest that PG-gel loaded with CM-UCMSCs effectively inhibits ovarian tissue fibrosis during *in vitro* culture. A schematic diagram of the ovarian tissue *in vitro* culture is shown in Figure 2F.

### 3.3 PG-gel loaded with CM-UCMSCs suppresses apoptosis of vitrified–thawed ovaries cultured *in vitro*

**FIGURE 3:**
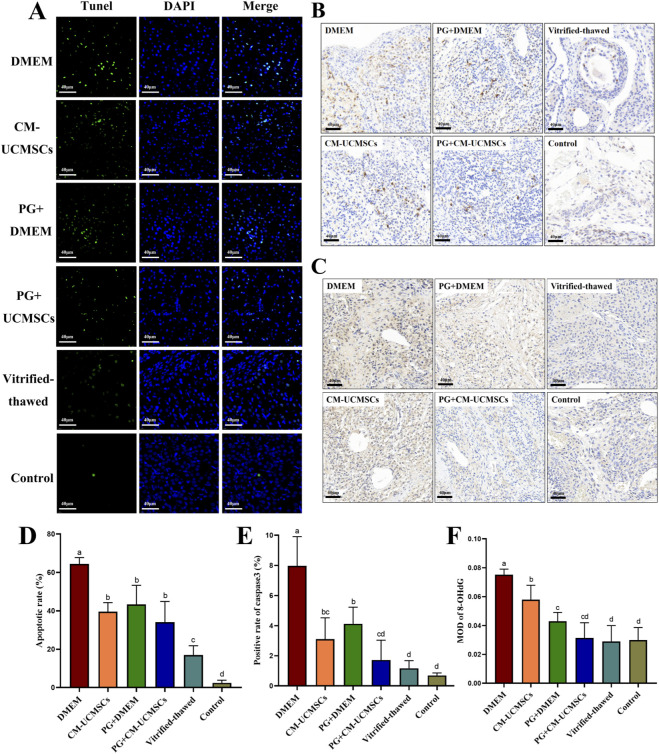
PG-gel loaded with CM-UCMSCs reduced apoptosis in vitrified–thawed ovaries cultured *in vitro*. Representative images showed apoptosis in the six groups by TUNEL staining (**(A)**, green indicates apoptotic signals) and immunohistochemical staining for caspase-3 **(B)** and 8-OHdG **(C)** (brownish-yellow indicates apoptotic signals). Scale bar = 40 µm. Quantitative results include apoptotic rate by TUNEL assay **(D)**, caspase-3 positive rate **(E)**, and mean optical density (MOD) of 8-OHdG **(F)**. Data are mean ± SD, n = 5 (biological replicates). Statistical differences (P < 0.05) were indicated by lowercase letters: bars sharing the same letter were not significantly different, while those without the same letter differ significantly.

Apoptosis in vitrified–thawed ovaries was evaluated with the TUNEL assay (Figure 3A), and TUNEL-positive cells were counted to calculate apoptosis rates. The four groups cultured *in vitro* after vitrification and thawing all showed significantly higher apoptosis rates than the vitrified–thawed and control groups (Figure 3D). Among the cultured groups, the DMEM group had the highest apoptosis rate, with the PG + CM-UCMSCs group showing the lowest apoptosis rate (Figure 3D).

To further assess ovarian apoptosis, immunohistochemical staining was performed for the apoptosis marker caspase-3 (Figure 3B) and the DNA damage marker 8-OHdG (Figure 3C). Quantification was based on positive rates (Figure 3E) and MOD values (Figure 3F). Consistent with the TUNEL assay, the PG + CM-UCMSCs group showed the lowest caspase-3 positive rate and 8-OHdG MOD among the four *in vitro* cultured groups. Both were significantly lower than in the DMEM group and not significantly different from the vitrified–thawed and control groups.

These results indicate that CM-UCMSCs and PG-gel each exert inhibitory effects on ovarian apoptosis, while PG-gel loaded with CM-UCMSCs shows a synergistic effect, more effectively suppressing apoptosis in ovaries cultured *in vitro*.

### 3.4 PG-gel loaded with CM-UCMSCs facilitates glucose consumption of vitrified–thawed ovaries cultured *in vitro*

**FIGURE 4:**
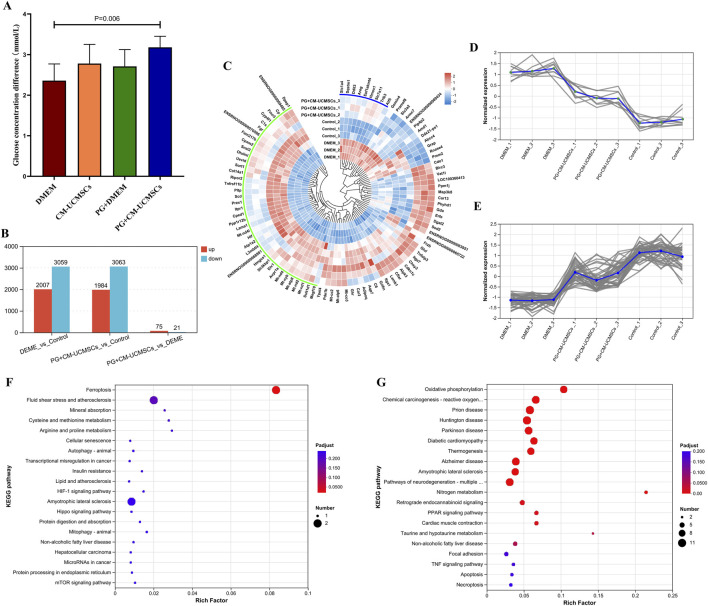
PG-gel loaded with CM-UCMSCs promoted energy metabolism and inhibited apoptosis in vitrified–thawed ovaries cultured *in vitro*. **(A)** Glucose concentration differences across four groups cultured *in vitro*. Data are mean ± SD, n = 5 (biological replicates). **(B)** Differential gene statistics (abscissa: pairwise sample comparisons; ordinate: number of DEGs). **(C)** Circular heatmap of DEGs (Cluster 1, blue arc; Cluster 2, green arc). **(D)** Expression of the nine genes in Cluster 1. **(E)** Expression of the 40 genes in Cluster 2. **(F)** Top 20 enriched KEGG pathways of downregulated DEGs (CM-UCMSCs vs. DMEM). **(G)** Top 20 enriched KEGG pathways of upregulated DEGs (CM-UCMSCs vs. DMEM).

To assess ovarian energy metabolism during *in vitro* culture, glucose concentration in the medium was measured before and after culture, and the difference (postculture minus preculture) was calculated. The PG + CM-UCMSCs group showed the largest difference, significantly higher than the DMEM group (p = 0.006) (Figure 4A). Differences in the CM-UCMSCs and PG + DMEM groups were intermediate and not significantly different from either the PG + CM-UCMSCs or DMEM groups (Figure 4A). These findings suggest that PG-gel loaded with CM-UCMSCs promotes glucose consumption and improves energy metabolism in vitrified–thawed ovaries cultured *in vitro*.

### 3.5 PG-gel loaded with CM-UCMSCs inhibits the expression of apoptosis-related genes and promotes the expression of genes linked to mitochondria and energy metabolism in vitrified–thawed ovaries cultured *in vitro*

To further investigate the protective mechanisms of PG-gel loaded with CM-UCMSCs in ovarian culture, the DMEM group, PG + CM-UCMSCs group, and control group were selected, with three parallel samples each, for RNA sequencing and bioinformatics analysis. Compared with the control group, the DMEM group showed 2,007 significantly upregulated and 3,059 significantly downregulated genes, while the PG + CM-UCMSCs group showed 1,984 significantly upregulated and 3,063 significantly downregulated genes (Figure 4B). In comparison with the DMEM group, the PG + CM-UCMSCs group had only 96 differentially expressed genes, comprising 75 upregulated and 21 downregulated (Figure 4B).

Clustering analysis was performed on the 96 DEGs between the PG + CM-UCMSCs and DMEM groups, dividing them into 10 clusters and visualizing expression patterns across the three groups with a circular heatmap (Figure 4C; Supplementary Table S1). Cluster 1 (Figure 4C, blue arc) contained nine genes closely associated with apoptosis. For example, elevated expression of Slc1a4 reduces intracellular serine levels, thereby inhibiting the PI3K/Akt/mTOR pathway and promoting GC apoptosis (Gu et al., 2025). Sqstm1, a molecular adaptor for selective macroautophagy (aggrephagy), participates in the autophagic degradation of ubiquitinated proteins and is positively correlated with cell death (Zhao et al., 2020). Ddit3 can activate transcription factor 5 (ATF5), inducing apoptosis (Teske et al., 2013). Ddit3 and Trib3 promote mitophagy and apoptosis through the ROS-JNK-DDit3 and Ddit3-Trib3-Akt-mTOR signaling axes (Huang et al., 2021). Hmox1 is strongly associated with apoptosis, particularly ferroptosis (Zheng et al., 2023). Expression of these apoptosis-related genes was highest in the DMEM group, intermediate in the PG + CM-UCMSCs group, and lowest in the control group (Figure 4D).

Cluster 2 (Figure 4C, green arc) comprised 40 genes. Among them, Mt-atp8, Mt-nd1, Mt-nd2, Mt-nd4, and Mt-cyb are all associated with ATP synthesis and respiratory chain function (Gencer Öncül et al., 2022). HIFs, a family of transcription factors, regulate the hypoxic stress response by enhancing glycolysis to maintain ATP levels under low oxygen (Zhao et al., 2024). Expression of these mitochondrial and energy metabolism–related genes was lowest in the DMEM group, intermediate in the PG + CM-UCMSCs group, and highest in the control group (Figure 4E).

KEGG enrichment analysis revealed that, compared with the PG + CM-UCMSCs group, genes significantly upregulated in the DMEM group were enriched in pathways such as ferroptosis and mTOR signaling (Figure 4F), which may regulate apoptosis. Conversely, significantly downregulated genes were enriched in metabolic pathways including oxidative phosphorylation, nitrogen metabolism, taurine and hypotaurine metabolism, and PPAR signaling (Figure 4G).

These findings suggest that during *in vitro* culture of vitrified–thawed ovaries, PG-gel loaded with CM-UCMSCs inhibits the expression of apoptosis-related genes and promotes the expression of genes linked to mitochondria and energy metabolism, thereby supporting ovarian function.

## 4 Discussion

OTCT, a key approach for female fertility preservation, faces major clinical challenges, including follicle loss, fibrosis, oxidative stress, and apoptosis caused by early post-transplantation ischemia and hypoxia (Dolmans et al., 2021; Sonmezer and Oktay, 2010). These factors greatly limit its clinical efficacy. In this study, we discovered that combining PG-gel with CM-UCMSCs markedly reduces damage and improves function in cryopreserved–thawed ovarian tissue during *in vitro* culture, showing strong potential for clinical application.

Glutathione is a key antioxidant in the human body (Elder et al., 2022). NAC, a derivative of L-cysteine, serves as a glutathione precursor and protects cells from free radical–induced damage through its sulfhydryl groups, making it a promising therapeutic antioxidant and chelating agent (Liu et al., 2022; Wu et al., 2020). After NAC modification, gelatin was converted into NAC-gelatin bearing thiol groups. NAC-gelatin was then combined with PEG-SS to form PG-gel, resulting in a safe, effective antioxidant biomaterial. When loaded with CM-UCMSCs, PG-gel exhibited a porous structure that facilitated the sustained release of bioactive factors. Thus, CM-UCMSCs–loaded PG-gel offers a promising strategy for improving cryopreserved ovarian transplantation protocols.

Our study demonstrated that PG-gel combined with CM-UCMSCs significantly reduces unhealthy follicle rates and exhibits strong antifibrotic, antiapoptotic, and antioxidative effects. Histological analysis showed that, compared with the DMEM group, the PG + CM-UCMSCs group had a 17.3% lower follicular abnormality rate (P = 0.012), a 10.9% decrease in fibrotic area (P = 0.011), a 30.3% reduction in apoptosis rate (TUNEL assay) (P < 0.001), a 6.8% decrease in caspase-3–positive cells (P < 0.001), and a 0.04 reduction in 8-OHdG MOD (P < 0.001). These effects may be attributed to the abundant bioactive factors in CM-UCMSCs, including vascular endothelial growth factor (VEGF), insulin-like growth factor-1 (IGF-1), hepatocyte growth factor, epidermal growth factor, and other cytokines that promote tissue regeneration, reduce inflammation and oxidative stress, inhibit apoptosis, and exert antifibrotic effects (Li et al., 2015; Xie et al., 2020). The sustained-release properties of PG-gel may further extend the activity of CM-UCMSC-derived factors, providing prolonged protection and maintaining follicular microenvironment homeostasis. Notably, PG-gel itself shows strong antioxidative activity: it suppresses collagen deposition by limiting oxidative stress–induced fibroblast activation, enhancing antifibrotic effects, and it mitigates oxidative damage by reducing ROS levels.

Energy metabolism is closely linked to cell survival and is a critical indicator of physiological function (Shen et al., 2015). Our results showed that PG-gel loaded with CM-UCMSCs significantly increased glucose consumption during *in vitro* culture of vitrified–thawed ovaries (P = 0.006), indicating improved ovarian energy metabolism. RNA sequencing further clarified the mechanisms. The system downregulated apoptosis-related genes such as Slc1a4, Sqstm1, Ddit3, and Trib3, which promote GC apoptosis through pathways including PI3K/Akt/mTOR and autophagy-dependent cell death (Gu et al., 2025; Huang et al., 2021; Zhao et al., 2020). It also upregulated mitochondrial function–related genes, such as Mt-atp8, Mt-nd1, Mt-nd2, Mt-nd4, and Mt-cyb, among others. KEGG analysis showed enrichment in metabolic pathways such as oxidative phosphorylation and PPAR signaling. These findings suggest that PG-gel loaded with CM-UCMSCs synergistically regulates energy metabolism and apoptosis pathways. Active factors in CM-UCMSCs (e.g., VEGF and IGF-1) not only enhance glycolysis and mitochondrial function to sustain ATP supply but also inhibit apoptosis signaling axes such as ROS-JNK-Ddit3, thereby exerting dual protective effects during *in vitro* culture of vitrified–thawed ovaries.

Research on ischemic–hypoxic injury after OTCT mainly focuses on two areas: promoting vascular reconstruction and reducing oxidative stress in ovaries (Cacciottola et al., 2021). Studies have shown that adding proangiogenic factors, antioxidants, hormones, or stem cells can effectively mitigate ovarian damage after OTCT (Cacciottola et al., 2021; Damous et al., 2018). However, these approaches face two limitations: first, the mechanisms of action require further validation; second, systemic administration results in insufficient ovarian drug concentration, while local administration cannot sustain effective levels due to rapid absorption. Repeated surgical delivery into the abdominal cavity is not clinically feasible, limiting therapeutic efficacy.

This study innovatively combined PG-gel with CM-UCMSCs for *in vitro* culture of vitrified–thawed ovarian tissue, demonstrating that the system protects follicles, inhibits fibrosis, and reduces oxidative damage, thereby improving ovarian function and providing an effective solution to these challenges. Although the synthesis and fundamental properties of PG-gel have been established in our team’s previous work, this study further optimized its performance and constructed a CM-UCMSCs-hydrogel composite system, which was successfully applied for the first time to the specific and complex biomedical scenario of *in vitro* culture and functional repair of vitrified-thawed ovarian tissue. This study not only provides a novel technical strategy for optimizing ovarian tissue before transplantation but also offers important insights for cryopreservation and regenerative medicine research in other organs.

Several limitations of this study must be acknowledged. First, although changes in glucose levels suggest that the combination of PG-gel with CM-UCMSCs may enhance energy metabolism, this indicator alone is insufficient to fully demonstrate an overall improvement in metabolic function. Direct evidence would require systematic analysis of downstream metabolites such as lactate, pyruvate, ATP, and succinate. Second, the absence of transcriptome sequencing for the CM-UCMSCs group without hydrogel prevents the distinction of the hydrogel’s independent effects at the transcriptional level, and thus the current conclusions primarily reflect the overall impact of the composite system. Future work will integrate metabolomic and comparative transcriptomic analyses to strengthen the evidence supporting the energy metabolism mechanism and to further clarify the specific role of the hydrogel in regulating cellular function. Additionally, this study employed a static culture system to validate the preliminary efficacy of the composite system. While this method effectively controls variables and facilitates mechanistic analysis, it does not replicate the dynamic *in vivo* microenvironment.

This study confirms through *in vitro* experiments that PG-gel loaded with CM-UCMSCs system exerts significant protective effects on vitrified-thawed ovarian tissue, offering a feasible and promising strategy for its clinical translation. The system possesses key characteristics such as controllable degradation, sustained release of active factors, and modulation of the microenvironment, demonstrating strong potential for clinical adaptation. Furthermore, the current research lays an important foundation for subsequent *in vivo* studies: the mechanisms through which it exerts protection via pathways such as antioxidant and anti-fibrotic effects have been preliminarily elucidated, providing crucial mechanistic guidance for future *in vivo* investigations. Future studies should examine the long-term effects of PG-gel loaded with CM-UCMSCs after *in vivo* transplantation and further clarify molecular mechanisms, particularly the roles of specific signaling pathways. Ultimately, these steps are critical for translating this promising strategy into a clinically viable therapy for restoring fertility and ovarian endocrine function.

## 5 Conclusion

This study demonstrated that combining PG-gel with CM-UCMSCs exerted significant protective effects in the *in vitro* culture of vitrified–thawed ovarian tissue, including reduced follicular abnormalities, inhibited fibrosis, decreased apoptosis, and alleviated oxidative stress. The underlying mechanisms may involve regulation of apoptosis-related gene expression, improved mitochondrial function, and enhanced energy metabolism. This innovative culture system offers a promising technological platform and theoretical basis for advancing female fertility preservation.

## Data Availability

The data presented in the study are deposited in the NCBI-SRA repository, accession number PRJNA1327384.
